# Modulation of Urea Transport Attenuates TLR2-Mediated Microglial Activation and Upregulates Microglial Metabolism In Vitro

**DOI:** 10.3390/metabo14110634

**Published:** 2024-11-17

**Authors:** Najlaa A. Al-Thani, Dylan Zinck, Gavin S. Stewart, Derek A. Costello

**Affiliations:** 1 School of Biomolecular and Biomedical Science, University College Dublin, D04 V1W8 Dublin, Ireland; najlaa.al-thani@ucdconnect.ie (N.A.A.-T.); dylan.zinck@ucd.ie (D.Z.); 2 UCD Conway Institute, University College Dublin, D04 V1W8 Dublin, Ireland; 3 School of Biology and Environmental Science, University College Dublin, D04 V1W8 Dublin, Ireland; gavin.stewart@ucd.ie

**Keywords:** UT-B, urea, nitric oxide, lipoteichoic acid, neuroinflammation, oxidative stress, 6-OHDA, tBHP

## Abstract

**Background:** Alzheimer’s disease (AD) is a neurodegenerative disorder traditionally characterised by the presence of amyloid beta (Aβ) plaques and neurofibrillary tau tangles in the brain. However, emerging research has highlighted additional metabolic hallmarks of AD pathology. These include the metabolic reprogramming of microglia in favour of glycolysis over oxidative phosphorylation. This shift is attributed to an ‘M1′-like pro-inflammatory phenotype, which exacerbates neuroinflammation and contributes to neuronal damage. The urea cycle also presents as an altered metabolic pathway in AD, due to elevated urea levels and altered expression of urea cycle enzymes, metabolites, and transporters in the brain. However, to date, these changes remain largely unexplored. **Methods:** This study focuses on understanding the effects of extracellular urea and urea transporter-B (UT-B) inhibition on inflammatory changes in lipoteichoic acid (LTA)-stimulated BV2 microglia and on the viability of SH-SY5Y neuronal cells under oxidative stress and neurotoxic conditions. **Results:** In BV2 microglia, UT-B inhibition demonstrated a notable anti-inflammatory effect by reducing the formation of nitric oxide (NO) and the expression of tumour necrosis factor α (TNFα) and CCL2 in response to stimulation with the toll-like receptor (TLR)2 agonist, lipoteichoic acid (LTA). This was accompanied by a reduction in extracellular urea and upregulation of UT-B expression. The application of exogenous urea was also shown to mediate the inflammatory profile of BV2 cells in a similar manner but had only a modest impact on UT-B expression. While exposure to LTA alone did not alter the microglial metabolic profile, inhibition of UT-B upregulated the expression of genes associated with both glycolysis and fatty acid oxidation. Conversely, neither increased extracellular urea nor UT-B inhibition had a significant impact on cell viability or cytotoxicity in SH-SY5Y neurones exposed to oxidative stressors tert-butyl hydroperoxide (t-BHP) and 6-hydroxydopamine (6-OHDA). **Conclusions:** This study further highlights the involvement of urea transport in regulating the neuroinflammation associated with AD. Moreover, we reveal a novel role for UT-B in maintaining microglial metabolic homeostasis. Taken together, these findings contribute supporting evidence to the regulation of UT-B as a therapeutic target for intervention into neuroinflammatory and neurodegenerative disease.

## 1. Introduction

Alzheimer’s disease (AD) is the leading form of dementia worldwide and the most common neurodegenerative disease [1]. Classical hallmarks of AD include neurofibrillary tau tangles, formed from hyperphosphorylated tau filaments, and amyloid beta (Aβ) plaques, formed from irregular cleavage of the amyloid precursor protein (APP) [2,3,4,5,6]. Pathology within the AD brain typically begins within the hippocampal region, resulting in cognitive decline [7,8,9]. However, this initiates a cascade of events involving chronic microglial activation, astrocytic reactivity, and oxidative and excitotoxic stress, leading to progressive neurodegeneration [4]. We, among others, have previously highlighted the critical role of microglial inflammatory pathways in mediating the neuronal dysfunction typical of conditions such as AD [10,11,12].

In recent years, metabolic hallmarks of AD pathology have been revealed, which has altered our understanding of AD as a late-age neurological disorder to a systemic middle-age metabolic disorder manifesting in the brain. For example, large-scale metabolic epidemiological studies have ascribed roles for impaired insulin secretion and glucose metabolism in contributing to the risk of AD among individuals with obesity and type-2 diabetes melitus (T2DM) [13,14]. This is likely due to the direct influence of metabolic disturbance on inflammatory function [15]. Furthermore, an emerging body of evidence has begun to characterise the dysfunctional microglia in the AD brain by the reprogramming of their metabolic profile. Recent studies have revealed that impaired glucose metabolism accompanies the pro-inflammatory ‘M1’-like microglial phenotype following Aβ exposure, which presents as a bias towards glycolysis rather than oxidative phosphorylation [16,17].

Dysregulation of the urea cycle and urea trafficking in the brain has also been reported in a number of neurodegenerative conditions, including AD [18,19,20,21,22,23], thus revealing further perturbation of cellular metabolic function. In the healthy brain, the urea cycle converts toxic ammonia into less toxic urea [24]. However, excess accumulation of both ammonia and urea has been detected in the brain during neurodegenerative disease, including AD [25,26,27], highlighting a disruption to this process. Indeed, this is true in multiple regions of the AD brain [27], directly implicating alteration of the urea cycle in the disease pathology. Although traditionally ascribed as a function of the liver and kidneys, the detection of key urea cycle enzymes, metabolites, and transporters in the brain points towards a functional urea cycle that plays a major role in maintaining neuronal and glial homeostasis [28,29,30,31,32,33].

Urea transporter-B (UT-B) has been identified as the primary urea transporter in the brain [34,35,36]. Moreover, upregulation of its expression is routinely identified in AD, as well as with age and other neurodegenerative disorders [22,30,36,37]. The role of UT-B in the brain was initially assigned to astrocytes and ependymal cells, in which it is most abundantly expressed [38]. However, our previous findings also revealed a role for UT-B in the regulation of inflammatory mediators from microglia and neuronal cells [39]. It is still not understood whether urea accumulation is a causative effect or a consequence of AD pathology. Due to the relative paucity of information on the regulation of urea production and trafficking in the central nervous system (CNS), the precise role of the urea cycle in the maintenance of neuronal integrity remains to be elucidated. This study sought to build upon our current knowledge by interrogating the impact of urea and urea trafficking on microglial function and neurodegeneration. We aimed to better understand the relationship between urea and microglial activation under conditions that mimic AD-like inflammation. In addition, we evaluated the potential role of urea in regulating neurotoxicity. Our compelling findings highlight a novel role for urea transport in maintaining microglial metabolic homeostasis. Moreover, we contribute to existing evidence in support of UT-B as a novel therapeutic target for neuroinflammatory diseases, including AD.

## 2. Materials and Methods

### 2.1. Cell Culture and Treatment

Cultured murine BV2 cells (kindly gifted by Prof. M. Lynch, TCD) and human SH-SY5Y cells (kindly gifted by Prof. M. Scott, UCD) were used to model microglia and neurones, respectively. Cells were grown in Dulbecco’s modified Eagle’s medium/Ham’s F-12 50/50 mix with L-glutamine (DMEM/F-12; Corning, USA), containing 10% *v*/*v* heat-inactivated Foetal Bovine Serum (FBS) and 1% Penicillin-Streptomycin (Gibco, Waltham, MA, USA), as previously described [11,39]. Cells were maintained in a sterile, humidified environment at 37 °C with 5% CO_2_. For treatment, cells were plated in 24-well (BV2) or 96-well (SH-SY5Y) tissue culture plates at a density of 1.5 × 10^5^ and 1 × 10^4^ cells/well, respectively. Pharmacological treatments were applied in FBS-free DMEM/F-12 in order to minimise exogenous urea contamination. Cells were incubated with lipoteichoic acid (LTA; 5 µg/mL; Sigma-Aldrich, Dorset, UK) in the presence and absence of urea (5, 7.5, 10, and 20 mM; Sigma-Aldrich, UK) or the selective UT-B inhibitor, UT-B-IN-1/UTB_inh_-14 (UT-Bi; 100 nM; [39]; MedChemExpress, Monmouth Junction, NJ, USA). In a separate set of experiments, urea or UT-Bi were co-applied to SH-SY5Y cells with oxidative stressors tert-butyl hydroperoxide (tBHP; 200 µM) or 6-hydroxydopamine (6-OHDA; 180 µM; Sigma-Aldrich, Dorset, UK). All treatments were applied to duplicate (BV2) or triplicate (SH-SY5Y) wells and incubated for 24 h.

#### Analysis of Nitrite, Urea, TNFα, and CCL2

Following treatment, supernatant was removed from BV2 cells and stored at −20 °C for later analysis of nitrite, urea, and TNFα concentration. The supernatant was analysed for the formation of the stable nitric oxide (NO) metabolite nitrite using a Griess assay (Sigma-Aldrich, Dorset, UK), as previously described [11,12]. Results were evaluated against a standard curve prepared with serial dilutions of NaNO_2_ (Sigma-Aldrich, Dorset, UK). Urea concentration was measured using a colorimetric Urea Microplate Assay kit (Cohesion Biosciences, London, UK), in accordance with the manufacturer’s instructions. The supernatant concentration of TNFα and CCL2 was determined using an enzyme-linked immunosorbent assay (ELISA; Invitrogen, Paisley, UK), according to the manufacturer’s guidelines. Results were determined as colorimetric changes read at wavelengths of 540 nm (Griess), 620 nm (urea), and 450 nm (TNFα, CCL2) using a SpectraMax M3 microplate reader and SoftMax Pro 6.2.1 software (Molecular Devices, San Jose, CA, USA). Values are expressed as a proportion of the control group within each independent experiment.

### 2.2. Gene Expression Analysis

RNA was extracted from BV2 cells following treatment using the E.Z.N.A.^®^ Total RNA Kit I (Omega Bio-tek, Norcross, GA, USA) in accordance with the manufacturer’s protocol. RNA was eluted in nuclease-free water (20 µL per sample) and quantified using the NanoDrop™ 2000 Spectrophotometer (Thermo Scientific, Waltham, MA, USA). Samples were stored at −80 °C. cDNA was prepared from 1 µg of RNA per sample by reverse transcriptase (RT)-PCR using the SuperScript^®^ III First-Strand Synthesis System (Invitrogen, Paisley, UK). cDNA was stored at −20 °C for subsequent analysis of gene expression.

qPCR was performed using the QuantStudio™ 7 Flex Real-Time PCR System to determine the expression of UT-B (*Slc14a1*) as well as genes associated with fatty acid oxidation (*Acadm* and *Cpt1a*) and glycolysis (*Hk3*, *Pfkfb1*, and *Pfkfb3*). cDNA templates were diluted (1:10) in nuclease-free water to a final volume of 10 µL qPCR reaction mixture containing SYBR™ Green PCR Master Mix (Applied Biosystems, Warrington, UK) and both forward and reverse primers (10 µM). The following primers were used: *Slc14a1*: forward 5′-CCCTCTTGCTTAGCCAACAGAG-3′, reverse 5′-CTGTCTTGGCTAAGCAAGAGG-3′ (Eurofins, Germany); *Acadm*: NM_007382; *Cpt1a*: NM_013495; *Hk3*: NM_001033245; *Pfkfb1*: NM_008824; and *Pfkfb3*: NM_001177752 (KiCqStart™ Primer Pairs, Sigma-Aldrich, Dorset, UK). The qPCR was carried out on a MicroAmp™ Optical 384-well reaction plate (Applied Biosystems, Warrington, UK), with non-template negative controls and melt curve analysis included in each run. Relative gene expression was calculated relative to the expression of the housekeeping gene glyceraldehyde 3-phosphate dehydrogenase (*Gapdh*) using the comparative Ct (ΔΔCt) method.

### 2.3. Cell Viability and Cytotoxicity Analysis

The viability of SH-SY5Y cells was determined in response to tBHP and 6-OHDA using the Cell Counting Kit-8 (CCK-8; Dojindo Laboratories, Rockville, MD, USA), in accordance with the manufacturer’s guidelines. In brief, supernatant was removed from SH-SY5Y cells following treatment and replaced with DMEM/F-12 containing 10% CCK-8 reagent (100 µL/well). Plates were incubated for 1–2 h (37 °C, 5% CO_2_), and 50 µL of supernatant was transferred to a new 96-well plate. Absorbance was measured at 450 nm. Cytotoxicity was determined based on supernatant expression of lactate dehydrogenase (LDH) using the CyQUANT™ LDH Cytotoxicity Assay kit (Invitrogen, Paisley, UK). Following treatment, 50 µL of supernatant was transferred to a fresh 96-well plate, and an equal volume of the reaction buffer was added to each sample. A known concentration of LDH and control DMEM/F-12 media were used as a positive control and assay blank, respectively. The plate was incubated on an orbital shaker for 30 min at room temperature; the reaction was stopped by application of stop solution, and absorbance was read at 490 nm and 680 nm. Absorbances were measured using the SpectraMax M3 microplate reader and SoftMax Pro 6.2.1 software (Molecular Devices, San Jose, CA, USA). Cell viability and cytotoxicity were calculated as a percentage of the control group.

### 2.4. Statistical Analysis

Statistical comparisons were made using two-way analysis of variance (ANOVA) to assess the effects of two independent variables or one-way ANOVA to compare across multiple treatments. Post hoc Tukey’s analysis was carried out to assess differences between specific groups. Graphs and statistical analysis were carried out using GraphPad Prism 10 software (GraphPad, Boston, MA, USA). Statistical significance is represented as * *p* < 0.05, ** *p* < 0.01, *** *p* < 0.001, and **** *p* < 0.0001. Data are represented as mean ± SEM, compiled with individual replicates from a minimum of 3 independent experiments.

## 3. Results

### 3.1. Influence of Urea and UT-B Inhibition on TLR2-Mediated Microglial Activation

**Figure 1:**
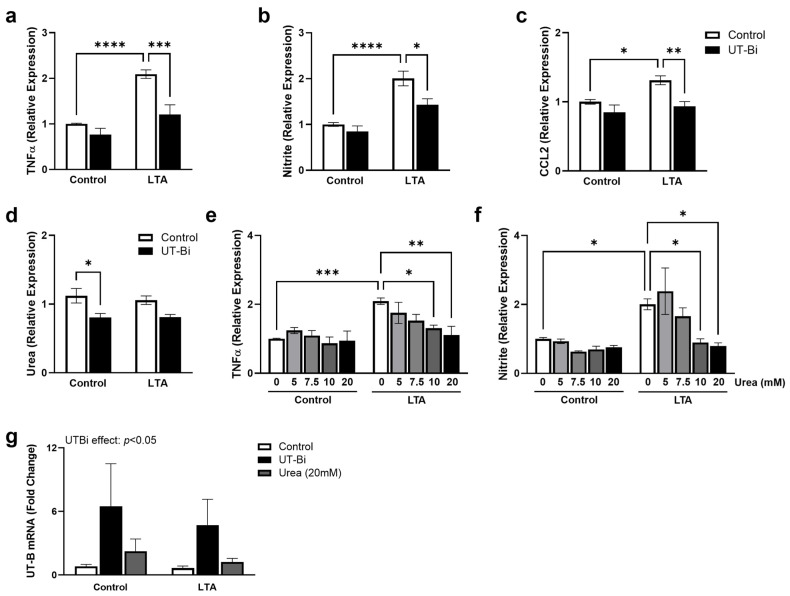
Inhibition of urea transport alleviates LTA-induced microglial activation. BV2 cells were exposed to LTA (5 μg/mL) for 24 h in the presence and absence of UT-Bi (100 nM) or urea (5, 7.5, 10, 20 mM). Supernatant expression of TNFa (**a**,**e**), formation of nitrite (**b**,**f**), CCL2 (**c**), and urea (**d**) was measured. Cellular expression of UT-B mRNA (*Slc14a1*; (**g**)) was assessed by quantitative PCR. Data are presented as mean ± SEM, relative to control values (n = 6–8 replicates from 3–4 independent experiments). Inset represents result of two-way ANOVA. * *p* < 0.05, ** *p* < 0.01, *** *p* < 0.001, **** *p* < 0.0001, two-way ANOVA followed by Tukey’s post hoc test.

In recent years, we, among others, have highlighted the prominent role of toll-like receptor (TLR)2 in mediating the inflammatory response and neuronal dysfunction associated with conditions such as AD [10,11,12]. Having previously reported that UT-B inhibition can alleviate lipopolysaccharide (LPS)-induced microglial activation [39], we now sought to explore the role of urea transport in regulating the TLR2-mediated response. BV2 cells are the most well-characterised in vitro model of murine microglia. In particular, their response to TLR stimulation has been well defined [12,39,40], revealing over 90% homology to that of primary microglial cells [41]. We have previously characterised the TLR2-mediated response in both primary microglia [10,42] and BV2 cells [11,12], revealing a clear commonality in their pro-inflammatory products. As expected, exposure to the TLR2 agonist lipoteichoic acid (LTA; 5 µg/mL) for 24 h significantly enhanced the supernatant concentration of TNFα (Figure 1a), formation of nitrite (Figure 1b), and expression of CCL2 (Figure 1c) from BV2 microglia, consistent with an ‘M1′-like phenotype (Figure 1a,b: *p* < 0.0001; Figure 1c: *p* < 0.05; Tukey’s post hoc test; n = 6–8 replicates from 3–4 independent experiments). Two-way ANOVA revealed a significant interaction between the effects of LTA and UT-Bi on TNFα (*p* < 0.05), illustrating that co-application with UT-Bi significantly attenuated the LTA-induced response (Figure 1a; *p* < 0.001, Tukey’s post hoc test; n = 6 replicates from 3 independent experiments). Similarly, the formation of nitrite was significantly lower in cells incubated with LTA and UT-Bi together, compared with those stimulated with LTA alone (Figure 1b: *p* < 0.05; Figure 1c: *p* < 0.01; Tukey’s post hoc test; n = 6–8 replicates from 3–4 independent experiments). Interestingly, the response to LTA was not accompanied by an alteration in extracellular urea concentration (Figure 1c). However, this was significantly reduced from control levels in the presence of UT-Bi (Figure 1c; *p* < 0.05, Tukey’s post hoc test; n = 6 replicates from 3 independent experiments). These findings demonstrate that inhibition of urea transport can alleviate the TLR2-mediated activation of microglia. Moreover, we provide important evidence supporting the role of UT-B in the cellular clearance of urea from microglia. We therefore sought to examine the impact of increasing extracellular urea on the regulation of the inflammatory response. BV2 microglia were exposed to extracellular urea (0–20 mM) in the presence and absence of LTA (5 µg/mL; 24 h). The application of urea at 10 and 20 mM significantly reduced the LTA-induced increase in TNFα (Figure 1d: *p* < 0.05, *p* < 0.01, respectively) and nitrite (Figure 1e; *p* < 0.05, Tukey’s post hoc test; n = 6–8 replicates from 3–4 independent experiments), in a similar manner to that seen in the presence of UT-Bi.

We have previously reported a modest, but significant, reduction in UT-B protein expression in microglia exposed to the TLR4 agonist LPS [39]. Surprisingly, here we found that exposure to LTA did not impact the expression of UT-B mRNA in BV2 cells, despite inducing a similar inflammatory response. Meanwhile, UT-B mRNA was significantly upregulated in the presence of the selective inhibitor UT-Bi, independently of challenge with LTA (Figure 1g; UTBi effect: *p* < 0.05, two-way ANOVA; n = 6–8 replicates from 3–4 independent experiments). This finding was mimicked to a lesser extent following exposure to 20 mM urea, although it did not reach statistical significance compared with the untreated controls (Figure 1g). Taken together, these findings indicate that preventing urea clearance, either through pharmacological inhibition of UT-B or altering the urea concentration gradient, can mitigate the pro-inflammatory activation of microglia.

### 3.2. UT-B Inhibition Upregulates Markers of Glycolytic Metabolism in Microglia

**Figure 2:**
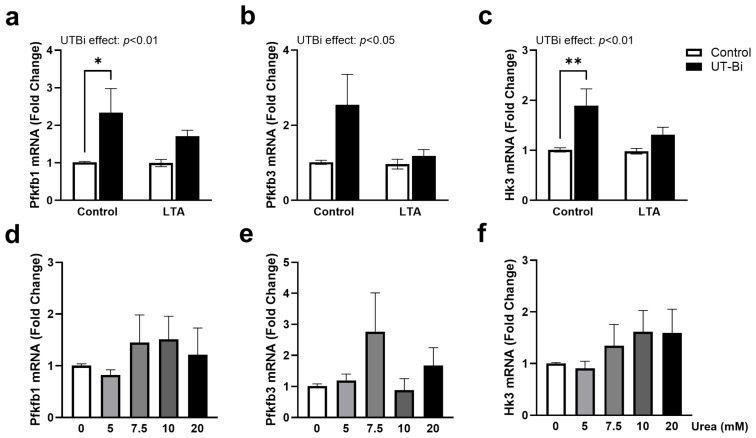
UT-Bi promotes expression of markers of glycolytic metabolism in microglia. BV2 cells were incubated with LTA (5 μg/mL) for 24 h in the presence and absence of UT-Bi (100 nM) or urea (5, 7.5, 10, 20 mM). Expression of *Pfkfb1* (**a**,**d**), *Pfkfb3* (**b**,**e**), and *Hk3* (**c**,**f**) mRNA was evaluated using qPCR with respect to expression of *Gapdh*. Data are presented as mean ± SEM, relative to control values (n = 5–6 independent experiments). Insets represent results of two-way ANOVA. * *p* < 0.05, ** *p* < 0.01, two-way ANOVA followed by Tukey’s post hoc test.

Recent research has begun to favour the characterisation of dysfunctional microglia based on their metabolic profile. Reprogramming towards glycolytic metabolism in favour of oxidative phosphorylation is known to accompany microglial polarisation to an M1 phenotype under AD-like conditions [43,44,45]. To assess the involvement of urea in regulating this activity, we examined the expression of key glycolytic enzymes in BV2 cells exposed to LTA (5 µg/mL) in the presence and absence of UT-Bi (100 nM) for 24 h. Gene expression analysis revealed no change in 6-phosphofructo-2-kinase/fructose-2,6-biphosphatase (*Pfkfb*)*1* and *Pfkfb3* in BV2 cells exposed to LTA (Figure 2a,b). Similarly, expression of hexokinase 3 (*Hk3*) was not altered in LTA-stimulated cells (Figure 2c). Unexpectedly, however, a two-way ANOVA revealed that each marker was significantly upregulated in response to UT-Bi compared with the controls (Figure 2a,c: *p* < 0.01; Figure 2b: *p* < 0.05; n = 5–6 independent experiments), indicative of enhanced glycolysis. Although not statistically significant, this increase appeared somewhat mitigated in the presence of LTA. A more variable and less profound increase in the expression of each marker was also apparent in the cells incubated with urea, although this did not reach statistical significance (Figure 2d–f). 

### 3.3. Inhibition of Urea Transport Promotes Markers of Oxidative Phosphorylation

**Figure 3:**
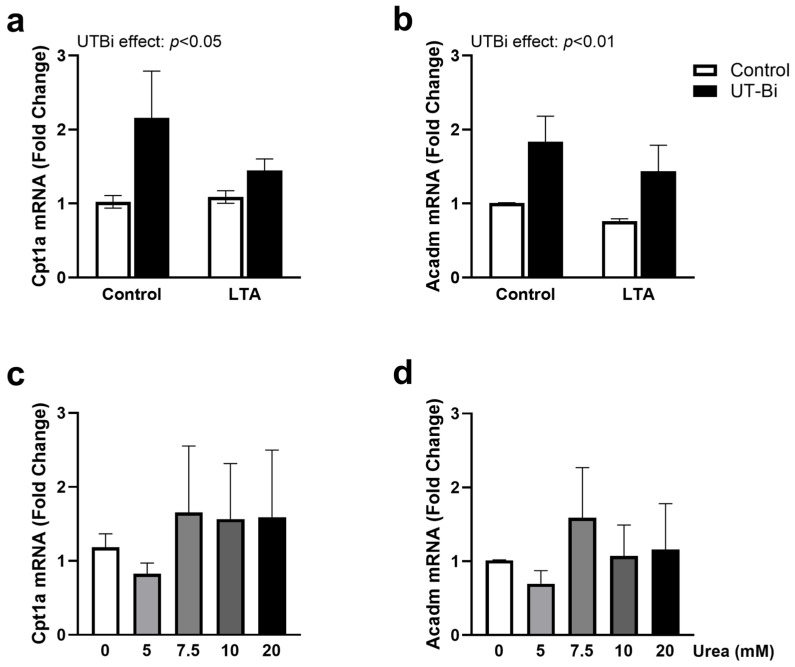
UT-Bi enhances markers of oxidative phosphorylation in microglia. BV2 cells were incubated with LTA (5 μg/mL) for 24 h in the presence and absence of UT-Bi (100 nM) or urea (5, 7.5, 10, 20 mM). mRNA expression of *Cpt1a* (**a**,**c**) and *Acadm* (**b**,**d**) was evaluated relative to *Gapdh* using qPCR. Data are presented as mean ± SEM, relative to control (n = 5–6 independent experiments). Insets represent results of two-way ANOVA.

To further explore the role of urea transport in regulating the relationship between glycolysis and oxidative phosphorylation in microglia, we assessed the expression of genes encoding the mitochondrial β-oxidation enzymes carnitine palmitoyltransferase 1A (*Cpt1a*) and acyl-CoA dehydrogenase (*Acadm*) [46]. Once again, these were not impacted by exposure to LTA (Figure 3a,b, respectively). Interestingly, however, a two-way ANOVA revealed a significant effect of UT-Bi in upregulating the expression of both *Cpt1a* (Figure 3a; UT-Bi effect: *p* < 0.05) and *Acadm* (Figure 3b; UT-Bi effect: *p* < 0.01; two-way ANOVA; n = 5–6 independent experiments). Incubation with urea did not consistently alter the expression of either *Cpt1a* (Figure 3c) or *Acadm* (Figure 3d) at any of the concentrations examined.

### 3.4. Oxidative Stress-Induced Neuronal Death Is Not Impacted by Urea or UT-B Inhibition

**Figure 4:**
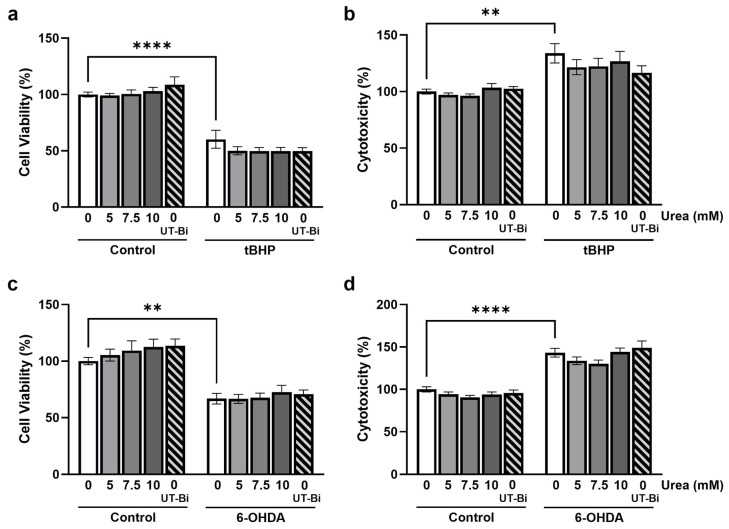
Inhibition of urea transport does not prevent oxidative stress-induced cell death. SH-SY5Y cells were treated with either tBHP (200 µM) or 6-OHDA (180 µM) for 24 h in the presence of either UT-Bi (100 nM) or urea (0–10 mM). Cell viability was evaluated by CCK-8 assay (**a**,**c**), and cytotoxicity was determined by supernatant expression of LDH (**b**,**d**). Data are expressed as mean ± SEM, as a percentage of control values (n = 9 replicates from 3 independent experiments). Statistical comparisons were determined by two-way ANOVA followed by Tukey’s multiple comparisons tests. ** *p* < 0.01, **** *p* < 0.0001.

Since chronic microglial activation accompanies neurodegeneration in the AD brain, we next sought to determine whether the modulation of urea trafficking can directly regulate neurotoxicity. Oxidative stress is widely recognised as a primary contributor to the neurodegeneration associated with AD [47]. Having previously identified that UT-Bi can modulate the release of inflammatory mediators from neuroblastoma cells [39], we used human SH-SY5Y neuroblastoma cells challenged with tBHP (200 µM; 24 h) to model oxidative stress-induced neurotoxicity. Cell death was determined by a significant reduction in cell viability (Figure 4a; *p* < 0.0001, Tukey’s post hoc test; n = 9 replicates from 3 independent experiments) coupled with an increase in supernatant concentration of LDH to signify cytotoxicity (Figure 4b; *p* < 0.01, Tukey’s post hoc test; n = 9 replicates from 3 independent experiments). Neither the exogenous application of urea nor UT-Bi alone significantly impacted cell viability (Figure 4a) or cytotoxicity (Figure 4b). Moreover, incubation with urea or UT-Bi did not modify tBHP-induced cell death (Figure 4a,b). To further evaluate the impact of urea on endogenously-produced reactive oxygen species, SH-SY5Y cells were exposed to the neurotoxin 6-OHDA, which promotes neuronal death through oxidative stress-mediated apoptosis [48]. Incubation with 6-OHDA (180 µM; 24 h) reliably attenuated cell viability and enhanced cytotoxicity (Figure 4c,d; *p* < 0.01, *p* < 0.0001, respectively; n = 9 replicates from 3 independent experiments). However, this was not significantly altered in the presence of urea or UT-Bi (Figure 4c,d). These findings indicate that regulation of urea transport does not directly influence neuronal oxidative stress.

## 4. Discussion

We have previously reported that inhibition of UT-B can alleviate LPS-induced microglial activation through reduction in cytokine and NO release [39]. Building on these findings, the current study offers new evidence that modulation of urea transport, either via increasing extracellular urea concentration or pharmacological inhibition of UT-B, can mitigate the TLR2-mediated inflammatory response. We further demonstrate that TLR2 stimulation is not directly associated with changes in UT-B expression in microglia. Preliminary results from the laboratory also indicate a similar lack of change in UT-B protein expression following LTA exposure. This is particularly interesting in light of our previous evidence of a reduction in UT-B in both microglia and neuronal cells in response to the TLR4 agonist LPS [39]. Both agonists stimulate similar signalling pathways leading to inflammation and neuronal dysfunction [42,49,50,51]. This suggests that the negative regulation of inflammatory changes by UT-B inhibition may be unrelated to the alteration in UT-B expression. One distinction in the signalling mediated by both receptors is the additional MyD88-independent pathway induced by LPS, leading to interferon-β production. Since this is known to mediate TLR4-induced apoptosis [51], it is plausible that the downregulation of UT-B in response to LPS is initiated through a similar mechanism, which does not arise following activation of TLR2. Increased expression of UT-B has routinely been observed in the AD brain [22,36,38]. Based on our findings, we can speculate that this increase may not represent an upregulation in microglial UT-B expression. Plausibly, more so, this could reflect an increase in the number of UT-B-expressing astrocytes, due to astrocytosis within the diseased brain, since astrocytes account for a significantly higher proportion of total brain cells and are known to express UT-B more abundantly than other cell types within the CNS [38,52]. Alternatively, we may speculate that a buildup of urea in AD promotes the glia expression of UT-B, as we report here following UT-B inhibition in vitro, which may be an attempt to compensate for impaired transport. This would infer that the change in UT-B is a consequence of the pathology rather than a causative factor.

Microglial TLR2 is well established as a receptor for the neurotoxins Aβ and α-synuclein [53,54], and we have previously highlighted its prevalent involvement in mediating Aβ-induced neuroinflammation and neuronal dysfunction [10,12]. ‘M1′-activated microglia are typically characterised by an upregulation in TNFα and NO production [55,56]. However, chronically dysfunctional microglia in the AD brain are identified by a metabolic shift in favour of glycolysis rather than oxidative phosphorylation. Previous reports have described changes in glycolytic markers such as *Pfkfb3* in microglia in vitro in response to IFNɣ [45]. Subsequent studies have described more robust metabolic changes when Aβ is applied in combination with IFNɣ [44] or LPS [43]. Our findings offer further support to existing evidence that multiple stimuli act synergistically to activate more complex cellular processes, such as inflammasome assembly [57], are necessary to modulate the microglial metabolic signature rather than TLR2 stimulation in isolation. We hypothesised that urea accumulation or disruption of urea transport may provide this synergistic stimulus to promote metabolic reprogramming in LTA-stimulated cells. However, extracellular urea does not appear to provide that synergistic response, further suggesting that it is likely a consequence rather than a cause of chronic microglial dysfunction. Surprisingly, however, inhibition of urea transport augmented markers of both glycolysis and oxidative phosphorylation in the absence of a change to inflammatory mediators. Moreover, this UT-Bi-induced expression of metabolic markers appeared dampened in LTA-stimulated cells. This indicates a response to UT-B inhibition that does not represent a change in microglial phenotype and does not adhere to a classical activation state. We can speculate that this overall enhancement in metabolic function is likely an attempt to compensate for the impaired urea regulation and points to a potential role for UT-B in maintaining homeostasis. It is likely, therefore, that microglia under inflammatory stress are less capable of evoking these same compensatory strategies. Of further interest was the observation that UT-Bi can upregulate *Cpt1a* expression in BV2 cells. As a key enzyme in the process of fatty acid oxidation, the role of *Cpt1a* is largely localised to astrocytes within the CNS. However, an upregulation in its activity has been reported to occur in the brain with age [58]. Our findings support the possibility that disruption of urea transport, as seen in disease states, may promote this activity in other cells such as microglia, which might not be evident under control conditions. This, in turn, could account for the increase observed in the aged brain and further point towards a potential role of UT-B in maintaining metabolic flux. Our study does not allow us to draw conclusions as to a definitive microglial phenotype induced by disruption of urea transport. Indeed, future work should explore the role of urea in regulating cellular respiration in microglia, both under control and activated conditions. This may offer further insight into the relationship between dyshomeostasis of urea trafficking and metabolic function.

In its role as a sensor for Gram-positive bacteria, TLR2 is also implicated in the pathogenesis of acute CNS infections such as bacterial meningitis [59]. Our findings propose that regulation of urea trafficking may offer an avenue for intervention against the TLR2-mediated inflammation associated with these conditions. Pathogens such as *Streptococci pneumoniae* and *agalactiae* are among the leading causes of bacterial meningitis, resulting in impairment of neuronal function and development and promoting susceptibility to neurodegenerative disease [59,60]. To a large extent, the neuronal dysfunction and neurodegeneration experienced by survivors of meningitis can be attributed to redox imbalance and oxidative stress [61]. Both the organic peroxide tBHP and the neurotoxin 6-OHDA are known to reliably promote neuronal apoptosis via the generation of reactive oxygen species [48,62]. Our findings reveal that regulation of urea transport does not restore neuronal integrity in response to direct challenge with neurotoxic oxidative stressors, suggesting that urea imbalance does not influence redox generation. We recognise that SH-SY5Y cells do not recapitulate the maturity and vulnerability of the neurones within the aged brain. Therefore, future studies might consider exploring the expression of UT-B and the impact of urea dysregulation on oxidative and excitotoxic death of mature or senescent neuronal cells to better model chronic neurodegenerative disease conditions. Our current findings also do not account for potential species-specific differences in UT-B, which may underlie the lack of response of our SH-SY5Y neurones. Expression of UT-B in the central nervous system was originally reported in cells [63] and tissue [34,64] from rat brain, in which we have further highlighted its ubiquitous expression [35]. It has since been identified in cells and tissues from mice [39,65], sheep [66], and humans [33], and is believed to act as the primary regulator of urea transport in the central nervous system [36]. Moreover, disruption in UT-B expression is reported in multiple human and mammalian disease models [20,66,67,68], suggesting that its role in brain pathology is not species-specific.

## 5. Conclusions

Taken together, our findings imply that the impact of urea imbalance in the inflamed brain, and indeed in neurodegenerative diseases, is likely to be mediated through regulation of microglial function rather than direct targeting of neurones. However, the mechanism through which urea and urea transport regulate the inflammatory response remains to be elucidated. Urea metabolism is closely coupled to that of NO due to their reliance on the use of L-arginine as a common substrate. Moreover, previous findings have consolidated this reciprocal relationship in the modulation of inflammation as well as neuronal and behavioural integrity [39,69]. The pivotal role played by NO in facilitating neurodegeneration has been well established [70,71]. Therefore, we hypothesise that the potential neurotherapeutic benefit of modulating urea trafficking may emerge indirectly through the manipulation of microglial NO generation and the consequent restoration of inflammatory homeostasis in the brain.

## Data Availability

Data are contained within the article.
